# Dr. Eve’s Reply to Dr. Otis’ Rejoinder

**Published:** 1868-10

**Authors:** Paul F. Eve

**Affiliations:** Nashville


### Correspondence.

### Dr. Eve's Reply to Dr. Otis' Rejoinder.

Mr. Editor:—Please accept my thanks for your kind remarks on the results of hip-joint amputations, made in the number of your valuable journal containing a lengthy communication signed George A. Otis, Ass't Surgeon and Brevet Lt. Col. U. S. Army. In my contribution to the history of this operation, contained in Vol. 18th Transactions of the American Medical Association, all intended was the expression of surprise as to how well the surgeons in the Southern army had done with it under the most disadvantageous circumstances; for by a comparison with the cases reported in Circular No. 6, issued from the Surgeon General's Office, but which is now known to be incomplete and erroneous, the results in the late Confederacy were, unexpectedly to me, very satisfactory. Cases developed since May, 1867, have changed these conclusions. You are right, Sir, in believing that I claim no superiority for Southern doctors, especially over those of the North, where most of us until recently, have been educated; for I well know how remarkably similar has been the rate of mortality in amputations in military surgery the world over. No invidious comparison was intended by my remarks. Dr. Otis in his rejoinder professes not only "to cast doubt upon many of my statements, but also to refute them;" he asserts that my cases are not sustained by the authors referred to, and that I have violated the courtesies of authorship. These I take to be pretty grave charges, and respectfully ask space in your journal for vindication.

It is certainly a question of no great importance to me, and it may be to the profession, what Dr. Otis may be pleased to consider a successful case of amputation at the hip-joint. When all the facts have been properly presented, each one will judge for himself. I believe I have the right, and now exercise it, in pronouncing Legoust's case a successful one, in opposition to all he or others may have written to the contrary; and I think this is the decision of the profession. All that was ever claimed in my contribution to the history of the hip-joint operations performed on the Southern side during the civil war, was that I acted *honestly.* As may be seen in the 18th vol. of the Transactions of the American Medical Association, "the particulars of each case have been derived as far as practicable from the operator himself, and his own language preserved; aiming to present the whole truth, every available means were employed, and in several instances the full history has been secured." What more justly could be done? If every fact furnished me by the authors of these cases were published, how could my statements be erroneous? And, moreover, if my deductions were not legitimate, the reader having their full history before him need not be deceived, even if that was my design. My statistics therefore of hip-joint amputations stand upon their own merits; *truthful* as far as could be obtained, and there they are left without further comment.

But I feel unwilling, Mr. Editor, for any one to make insinuations or cast aspersions upon me, especially when they are unmerited and can be easily exposed. This reply is provoked from me and I am acting purely on the defensive.

Dr. Otis says that he, and he alone, ("personally" is the word,) engaged me to collect the cases of hip-joint amputations in the Southern army; yet kindly admits that I regarded them designed for the use of the Medical Bureau of the Surgeon General. Fortunately the following extracts from a few of the many letters written me by Dr. Otis, will make this quite satisfactory as far as I am concerned: "Surgeon General's Office, Washington City, Feb'y 9th, 1867. I have the honor to inform you that, by the direction of the Surgeon General, I am engaged in compiling a manuscript on Amputations at the Hip-joint in Military Surgery. * * * Drs. Chisholm, Gaillard, Maury and others, have lent me their assistance. I have only been able to collect *three* examples. * * * I shall esteem it a great kindness if you will facilitate my inquiries, and which I feel great diffidence in trespassing upon your valuable time, your former kindness emboldens me to seek your assistance. March 20th, (in about a month, exclusive of the time for transmission of letters, he writes:) I am under the greatest obligation to you for your kind interest in aiding me to complete the records of hip-joint amputations during the war. April 19th, I have received your letters of the 11th and 12th of April, with the record of *thirteen cases* of amputations at the hip-joint. Gilmore's, Lay's, Grant's, Compton's and Felton's cases, *seven in all*, are entirely new to me. * * * *The Surgeon General desires me to express to you with his kindest regards, his appreciation of your valuable contributions to the* SURGICAL DATA OF HIS OFFICE. I hardly know how to classify *Gilmore's and Compton's successful cases.* (The reader will please remember that this is the language of Dr. Otis, who now assails me for publishing Dr. Gilmore's case a successful one, and here we have his own admission that it was so.) May 7th. You quite overwhelm me with your rich contributions, and I hardly know how to express my thanks. The new cases are of great interest. * * * I have been *delayed* in printing my paper, since, mainly by your kind assistance, I have improved the delay by the acquisition of so much interesting material. *Surgeon General's Office, Division of Surgical Records, May 7th,* 1867. *Respectfully returned to Prof. Paul F. Eve, with many thanks.* Oct. 22d. Thanks for your letter received. Having had the pleasure of reading it, I have sent it up to the Surgeon General."

In addition to these extracts, the fact is now mentioned that soon after my return to Nashville from the late Confederacy, I was otherwise the recipient of kindness from the Surgeon General of the U. S. Army, attributed as is believed to my having been in early life, the schoolmate of a distinguished Georgia Governor, who had, no doubt, presented me favorably to him, and hence in all probability, the application to me from his office through Dr. Otis to contribute what I could to the history of amputation at the hip-joint. I trust the reader is now prepared to decide whether I was more presumptuous in believing that I was working for the U. S. Army Medical Bureau, in collecting the cases of hip-joint amputation which had occurred in the Southern service, or Dr. Otis in flattering himself that I was engaged voluntarily in laboring for himself exclusively.

While acknowledging that I had furnished twenty cases of amputation at the hip-joint to the Surgeon General's office, *sixteen of which he had never heard of*, and claiming them for his own *personal use*, yet in the same paragraph, Dr. Otis insinuates that I violated the courtesies of authorship by publishing "these materials in the Transactions of the Medical Society of Tennessee and the Transactions of the American Medical Association, prior to the official publication of the bureau for which they were *ostensibly* collected." It might be well for him to be better informed before making such a railing accusation; he even italicised the names of the above medical corporations. Now it is not true that my contribution to the hip-joint operations was ever *presented*, or *read*, or *published*, by the Tennessee State Medical Society. The paper received by that body was the "Statistics of ninety cases of Urinary Calculus, 78 Bi-lateral, 3 Lateral, 3 Lithotrity, 2 Vaginal, 1 Urethral section, and 1 Dilatation." Nor was the article presented to the American Medical Association ever sent to Washington city for Dr. Otis or the Surgeon General. All they asked for or received were the cases as collected by me; and the imputation that I presented to and had published by the State Medical Society here, by the American Medical Association and the Army Medical Bureau, one and the same paper, is unequivocally an error. Any one by examining the proceedings of the two named societies and Circular No. 7, issued by the Surgeon General U. S. Army, July, 1867, will discover the fact, that in but one of them alone can my contribution to the history of the hip-joint operations be found. But this is not all; believing that my cases were collected for the use of the Army Medical Bureau, and as Dr. Otis had notified me again and again, the *delay* which had occurred in the contemplated publication from his office, I wrote and *obtained his permission* to do the very thing for which he now attempts to censure me. Here is his reply: "Surgeon General's Office, Washington City, March 27th, 1867. Dear Doctor: It would be very *unreasonable in me to object* to the reading of your contribution to the record of the hip-joint amputation at the meeting of your State Society. My memoir aims at completeness rather than originality, and *most of the cases included in it have already been in print.*" Moreover, it was six weeks after this that my paper was presented to the surgical section of the American Medical Association and by it ordered to be published. The 18th volume, in which it appeared as an original communication, is dated July, while Circular No. 7, containing only acknowledgments and references to the cases furnished by me, which are simply classified by Dr. Otis with others, collected by him from the whole country, is actually dated by himself, *June.* How, then, had I *published* these materials prior to his official publication? Did July come before

June in 1867? With these irrefutable facts before him, Dr. Otis has volunteered to accuse me of committing a breach of courtesy as an author, when the truth is, he not only gave me permission to read my paper before a medical association, but he actually published the cases prior to, or as soon as the proceedings of that body were printed, and after many acknowledged delays of publication on his part.

Another point of Dr. Otis is, that I published as a success, the case of Dr. J. T. Gilmore, formerly of the old army, now a Professor in the Mobile Medical College, which, he asserts, "the modest and conscientious operator, remembering that the patients of M. Legoust and Dr. Wier, died three or four months after operation, advances no such claims." The inference then, is, that no amputation at the hip-joint is to be admitted hereafter as one of unequivocal success, which has not survived beyond this period. Is the profession prepared to adopt this standard? If so, the statistics of this operation, amputation in general, must be reviewed; a thing now utterly impossible. But if as has heretofore been the custom to consider all cases successful when the *wound made by the operation has healed*, which has proved reasonable and judicious, then may we hope for satisfactory conclusions. In proof, however, that the operator, (Dr. Gilmore,) in the case above referred to *did claim it a success*, I quote his own published language now in the possession of Otis: "I heard," says he, "from his friends that *he recovered entirely and went home.* * * * I therefore regarded this, though *successful*, as not a fair criterion by which to judge of amputation at the hip-joint, especially a primary operation in military surgery." The 30th of last June, Dr. Gilmore wrote me from Mobile, "the chances are *that he recovered perfectly.*" This, too, is the very case which I have shown Dr. Otis himself admitted, April 19th, 1867, was *successful.* Will any one now believe that I am not sustained by my authority, or that Dr. Otis can prove what he has alleged on this point?

Again, he writes me that while I believe courtesy requires all who may cast doubts on an author's statements should notify the assailed party, he complains that he was subjected to the expense of purchasing my paper from the publisher. I have always been opposed to anonymous communications on scientific subjects, and know no good reason why doctors should thus attack each other in the dark. I love a man who has an open heart. Dr. Otis sent me his rejoinder, but I never read it until it was published. I very much regret he had to pay for so worthless an article as my pamphlet, but even here there must be an error. Mr. T. K. Collins of Philadelphia, publisher for the American Medical Association, declares most positively that he had no right to sell, and never sold a copy; and I am ready to make oath that I have supplied every one, even in Europe, who expressed a wish for the pamphlet, free of all expense. The publisher says Dr. Otis must have bought the volume of Transactions of the Association containing it, and not a copy of the paper which I had issued in an extra form.

In the concluding paragraph of Dr. Otis' rejoinder, he expresses the hope that the "field of science will not be invaded by partisan prejudice, and that to us will be left at least the republic of letters." Before he opened the correspondence with me in February, 1867, (without he once resided in Richmond, Va.,) I knew nothing of him. He then commenced addressing me as "Dear Sir," then "Dear Doctor," changing this to "My Dear Doctor," "My Dear Professor," but presume our correspondence has now ceased by his enclosing his rejoinder to the "Doctor." When at the Association last May in Washington, I called several times to see him, and found him to fail at every appointment made for me to meet the Surgeon General, a single remark made at that time when I last saw him satisfied me in regard to the bias of his mind. In alluding to the hip-joint amputation he said, "You Southern gentlemen have so much chivalry;" and this I hope we may never lose, always exercising that charity which relies in a brother's word of honor. "I question no man's veracity, as I will not submit my own to be called in question." To leave us the republic of letters and sciences is just now the prayer of the whole South. But, alas! what fields or republics of either kind whatsoever remain to us uninvaded? We read in the last *Presbyterian Index*, published in Mobile, that the entire faculty of the University of Alabama is composed of invaders from Ohio; and it states that one of these professors was beaten by a negro for door-keeper of the Legislature at Montgomery.

It has given me no pleasure, Mr. Editor, to make this expose. I love my profession and all connected with it. I believe Dr.

Otis is a worthy member of it, and I commend his talent, industry and perseverance. But since his full, free and it may be said extraordinary acknowledgments of my coöperation with him as the reporter of Circular No. 7 from the Surgeon General's office, a change has certainly come over him. But be the cause what it may I can safely rely for justice upon that profession which has done so much for me and honored me so far above my deserts.

PAUL F. EVE, M. D.

Nashville, 18th Sept., 1868.

